# Assessment of sleep disturbance in patients with Wilson’s disease

**DOI:** 10.1186/s12888-024-05493-w

**Published:** 2024-03-13

**Authors:** Zhengyang Wang, ZhiFei You

**Affiliations:** 1 Department of Neurology, The First Affiliated Hospital of Chongqing Medical University, Chongqing, China; 2 Department of Neurology, Taizhou Clinical Medical School of Nanjing Medical University, Jiangsu Taizhou People’s Hospital, 225300 Taizhou, China

**Keywords:** Wilson’s disease, Sleep disturbance, Neurological variant, Mood, Depression

## Abstract

**Background:**

Wilson’s disease (WD) is frequently manifested with anxiety, depression and sleep disturbance; this investigation aimed to elucidate these manifestations and identify the influencing factors of sleep disturbance.

**Methods:**

Sleep disturbance, anxiety and depression were compared in 42 WD and 40 age- and gender-matched healthy individuals. 27 individuals indicated a neurological form of the disease (NV), and 15 had a non-neurological variant (NNV).

**Results:**

This investigation revealed that the Parkinson’s disease sleep scale (PDSS) score of WD individuals was lower, whereas their Epworth Sleepiness Scale (ESS), Pittsburgh sleep quality index (PSQI), Hamilton Anxiety Scale (HAMA), and Hamilton Depression Scale (HAMD) scores were higher than the healthy individuals (*p* < 0.05). Furthermore, the WD subjects had markedly increased prevalence of poor sleep quality, anxiety, and depression than healthy individuals (*p* < 0.05). Subgroup analysis showed that NV subjects had significantly higher scores on the UWDRS, PSQI, HAMA, and HAMD scales than those in the NV group, as well as higher rates of EDS, anxiety, and depression (*p* < 0.05). In patients with sleep disturbance, we identified UWDRS, neurological variant, and depression as associated factors. The linear regression model demonstrated depression as the dominant risk factor.

**Conclusions:**

Depression is highly correlated with and is a determinant of sleep disturbance in WD patients.

## Introduction

Wilson’s disease (WD) is a rare hereditary neurometabolic caused by a mutation in ATP7B gene [1]. Then abnormal copper accumulation in the central nervous system, liver and other organs can lead to multiple clinical presentations. Neurological and hepatic manifestations are most common, but patients may also present with psychiatric, cognitive, or renal symptoms [2]. Main neurologic features are motor symptoms but non-motor symptoms are also frequent associating sleep disturbance, depression, anxiety, cognitive impairment, and autonomic disturbances [3].

Mood disorders, which are often under-recognized, are the most common psychiatric manifestations of WD. Comorbidity between WD and depression has been estimated in previous work at 20–60%, anxiety around 20% and sleep disturbance in around 40–80% [4, 5]. Depression and anxiety are common comorbidities of insomnia, especially depression, which could affect sleep quality in movement disorders such as Parkinson’s disease [6]. These may indicate that depression, while playing a significant role in sleep disturbance associated with WD. However, meaningful studies focusing on the potential link between anxiety, depression and poor sleep are scarce in the literature.

Therefore, the present work aims to elucidate and characterize anxiety, depression and sleep quality in WD patients using structured questionnaires and scales, and to compare them with healthy controls. We hypothesize that: (1) there is a marked association of depression with sleep disturbance and (2) sleep quality is more aberrant in individuals with severe depression.

## Materials and methods

### Population and methods

From 2015 to 2020, 42 WD individuals (27 males, 15 females, > 18 years old) who enrolled at the neurological clinics in the Shanghai Jiao Tong University School of Medicine were selected for this investigation based on the diagnostic criteria for Wilson’s disease of European Association for Study of Liver [7]. Furthermore, 40 age- and gender-matched healthy individuals (15 females and 25 males) were enrolled as control. Based on the previous classification, participants suffering from the neurological manifestations were categorized as a neurological variant (NV), and those with the chronic liver disease but without neurological manifestations were grouped as the non-neurological variant (NNV) [8].

To assess the neurological and functional assessment of WD patients, the Unified Wilson’s disease rating scale (UWDRS) was utilized [9]. The sleep quality and mood disorders of all the subjects were determined via the following questionnaires and scales: Parkinson’s disease Sleep Scale (PDSS) comprising 15 items [10], Pittsburgh sleep quality index (PSQI), a self-rated questionnaire, where the cut-off value of 5 depicts poor sleep quality [11], Epworth Sleepiness Scale (ESS) to assess the consequence of sleep disturbances in patient’s daily activity, a score of more than 10 denoted excessive daytime sleepiness (EDS) [12], and Hamilton Anxiety Scale (HAMA, 14 items), and Hamilton Depression Scale (HAMD, 17-item) to screen anxiety and depression respectively, a cut-off score of 7 was treated as abnormal [13, 14].

The Ethical board of the Jiangsu Taizhou People’s Hospital authorized this analysis as part of a primary program from the Shanghai Jiao Tong University School of Medicine (No. 2022-KY005, date of approval: 2022-01-24), and all the participants were first informed about the study before acquiring signed informed consent.

### Statistical analysis

The continuous variables that conform to a normal distribution were represented as mean ± standard deviation (SD) and the one-way ANOVA or Student’s t-test was used to compare the differences among/between groups. The continuous variables that do not conform to a normal distribution were presented as the median and interquartile range (IQR). The comparison among/between groups were finished by Kruskal-Wallis test or Mann-Whitney U test. The categorical variables were presented as frequency and percentage. Chi-square or Fisher test was used to compare the group differences. Relational questions were assessed by Spearman’s correlation coefficient and multiple linear regression analyses. The data were processed via SPSS 20.0, and *P* < 0.05 was deemed statistically important.

## Results

### Clinical manifestation in the diseased and the control cohort

Subject description and clinical and demographic features are described in Table 1. The WD group comprised 27 males and 15 females of 18 to 50 years of age (median 26 years), with the course of the disease being 2 months to 18 years (median 5 years), whereas the NNV group included 15 patients (8 males and 7 females). All patients received zinc salt treatment and were stabilized on a maintenance phase of therapy. It is suggested that zinc salts do not affect sleep quality; however, supporting data are lacking. Additionally, 40 healthy individuals, 25 males, and 15 females, 18 to 50 years of age (median 26 years), were included in the control group.

**Table 1:** Demographic and clinical characteristics

	Controls	Patients	***P***	NV	NNV	***P*** ^#^	***P*** ^##^
Sex (male/female)	25/15	27/15	1	19/8	8/7	0.566	0.442
Age (years)	26 (21, 33)	26 (22.5, 33)	0.950	27.15 ± 7.06	29.27 ± 9.08	0.865	0.406
Disease duration (years)	-	5 (2, 13.25)	-	5 (3, 14)	5 (1, 8)	-	0.222
UWDRS	-	10.5 (4, 39.25)	-	30 (10, 46)	3 (0, 5)	-	**< 0.001**
PDSS items							
Quality of sleep (PDSS1)	9 (8, 10)	8 (6, 9.25)	0.082	8 (5, 9)	9 (8, 10)	**0.041**	0.069
difficulty falling asleep (PDSS2)	10 (10, 10)	10 (8.75, 10)	**0.003**	10 (9, 10)	10 (8, 10)	**0.013**	0.903
difficulty staying asleep (PDSS3)	10 (10, 10)	10 (9.75, 10)	**0.004**	10 (8, 10)	10 (10, 10)	**0.001**	**0.030**
Restlessness of legs (PDSS4)	10 (8, 10)	10 (10, 10)	0.077	10 (9, 10)	10 (10, 10)	0.144	0.335
Fidgety in bed (PDSS5)	9 (8, 10)	9 (8, 10)	0.055	8 (5, 10)	9 (8, 10)	**0.046**	0.112
Distressing dreams (PDSS6)	9.5 (8, 10)	10 (8.75, 10)	0.942	10 (8, 10)	9 (9, 10)	0.990	0.832
Hallucination at night (PDSS7)	9 (9, 10)	9 (7.75, 10)	0.052	9 (6, 10)	9 (8, 10)	0.111	0.364
Nycturia (PDSS8)	9 (8.25, 10)	9 (5.75, 10)	0.334	9 (5, 10)	9 (7, 10)	0.407	0.470
Urine incontinence (PDSS9)	10 (10, 10)	10 (10, 10)	0.241	10 (10, 10)	10 (10, 10)	**0.044**	0.294
Numbness/tingling in legs (PDSS10)	10 (10, 10)	10 (9, 10)	0.050	10 (10, 10)	10 (9, 10)	**0.037**	0.165
Nocturnal cramps (PDSS11)	10 (10, 10)	10 (10, 10)	0.056	10 (10, 10)	10 (9, 10)	0.139	0.726
Painful posturing (PDSS 12)	10 (10, 10)	10 (10, 10)	0.447	10 (10, 10)	10 (10, 10)	0.662	0.413
Tremor on wakening (PDSS13)	10 (10, 10)	10 (10, 10)	**0.036**	10 (10, 10)	10 (10, 10)	0.082	0.770
Sleep refreshment (PDSS14)	8 (5.25, 10)	8 (5, 10)	0.224	8 (5, 10)	8 (5, 10)	0.450	0.756
Daytime dosing (PDSS15)	8 (8, 9.75)	8 (7, 10)	0.893	8 (7, 10)	9 (7, 10)	0.865	0.648
PDSS	135 (126, 141)	142 (134, 145)	**0.001**	132 (124, 138)	136 (130, 142)	**0.002**	0.133
PSQI	4 (3, 5)	5 (4, 7)	**< 0.001**	6 (4, 8)	4 (3, 6)	**< 0.001**	**0.023**
ESS	4 (2, 4)	5 (3.75, 8)	**0.044**	5.37 ± 3.14	5.67 ± 3.29	0.128	0.775
HAMA	1 (0, 1)	4 (2, 7)	**< 0.001**	4 (3, 8)	2 (1, 4)	**< 0.001**	**0.031**
HAMD	1 (0, 2)	5 (3, 8)	**< 0.001**	7 (4, 10)	3 (2, 6)	**< 0.001**	**0.048**

p: comparison between Controls and Patients;

*P*^#^: comparison among the three groups (Controls, NV, NNV);

*P*^##^: comparison between NV and NNV;

**Abbreviations:** Neurological variant (NV); Non-neurological variant (NNV); Unified Wilson’s disease rating scale (UWDRS); Parkinson’s disease Sleep Scale (PDSS); Pittsburgh sleep quality index (PSQI); Epworth Sleepiness Scale (ESS); Hamilton Anxiety Scale (HAMA); Hamilton Depression Scale (HAMD)

**Fig. 1:**
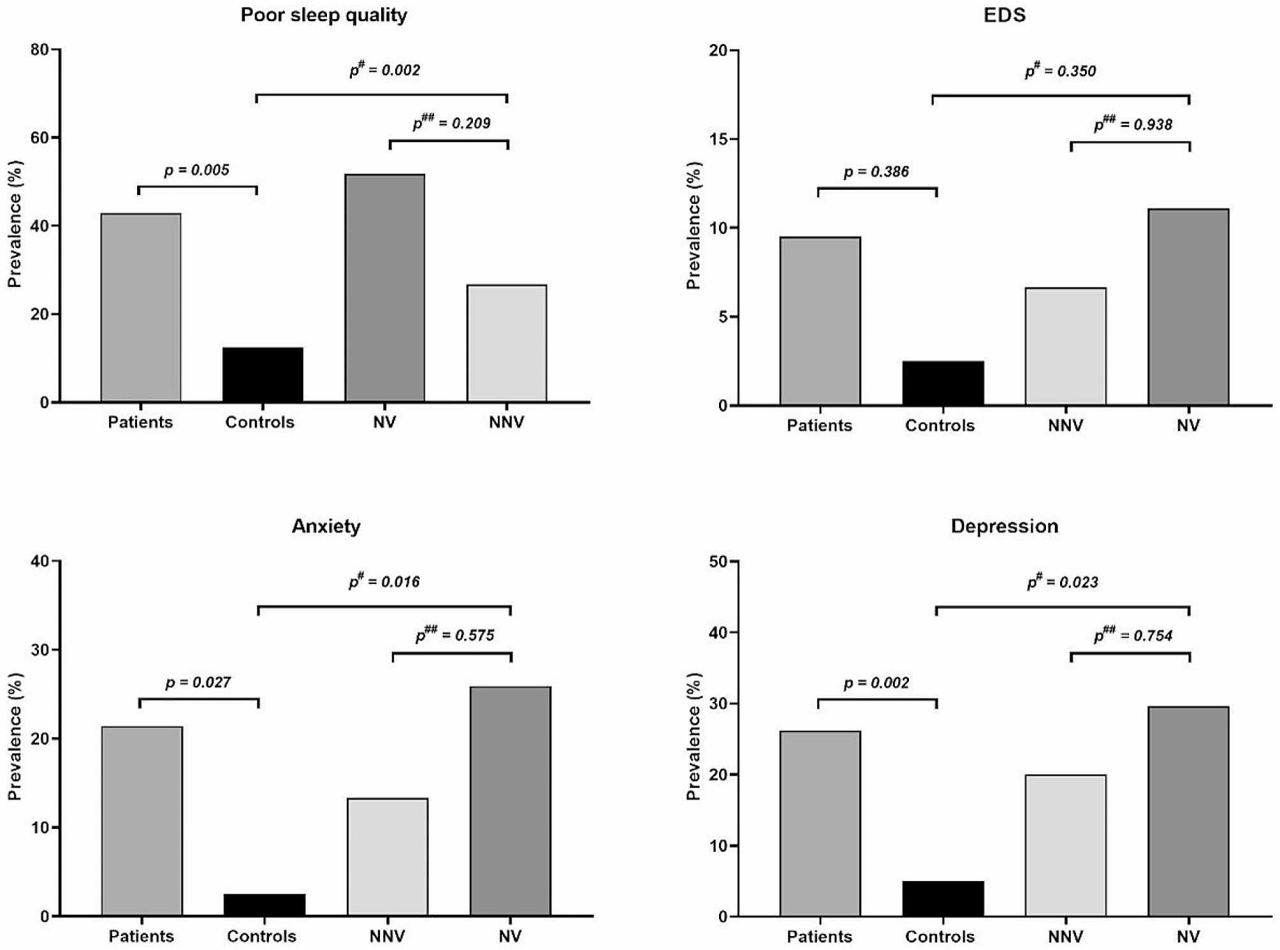
Comparison of anxiety, depression and sleep disturbance in patients with Wilson’s disease compared to controls

​The mean PDSS score of the healthy controls is substantially higher than that of the WD group (*p* = 0.001). The subtest scores, including difficulty falling asleep (*p* = 0.003), staying asleep (*p* = 0.004), and tremor on wakening (*p* = 0.036), were substantially reduced in the WD cohort than normal cohort (Table 1). The ESS, PSQI, HAMA, and HAMD scores were markedly lower in healthy individuals than in diseased individuals (*p* < 0.05). Participants with sleep disturbance and depression were more in the diseased cohort than in the control cohort, even though patients frequently took antidepressants. The prevalence of poor sleep quality, anxiety and depression was notably more in the WD group than in the controls (*p* < 0.05, Fig. 1).

The comparison between NV and NNV groups indicated that individuals with neurologic phenotype had notably increased UWDRS, PSQI, HAMA, and HAMD scores than the NNV group (*p* < 0.05). The PDSS subset score of difficulty staying asleep (*p* = 0.030) was substantially higher than the NNV subjects. Upon comparing all three groups (NV, NNV, and Control), marked differences were observed for PDSS (*p* = 0.002), PSQI (*p* < 0.001), HAMA (*p* < 0.001), and HAMD (*p* < 0.001) scores. Subgroup analysis indicated reduced PDSS and elevated HAMA and HAMD scores among NV and NNV subjects than in controls. In contrast, PSQI scores were substantially elevated in NV subjects than in healthy controls (*p* < 0.05). Our results demonstrated that the prevalence of poor sleep quality, EDS, anxiety, and depression had an enhanced trend in the NV group, but the difference was not statistically significant (*p* > 0.05, Fig. 1).

### Correlation and regression analyses

The association of sleep disturbance with psychopathological and neurological variants and disease severity was assessed (Table 2). Spearman’s correlation revealed a positive association of UWDRS with neurological variant (*r* = 0.758, *p* < 0.001), HAMA (*r* = 0.407, *p* = 0.008), and PSQI scores (*r* = 0.327, *p* = 0.035). Moreover, the neurological variant of the disease has a positive correlation with HAMA (*r* = 0.336, *p* = 0.030), HAMD (*r* = 0.309, *p* = 0.046), and PSQI (*r* = 0.354, *p* = 0.021) scores. A strong positive relationship between HAMA and HAMD scores (*r* = 0.488, *p* = 0.001) was observed; however, no correlation was discovered between HAMA and PSQI, ESS, and PDSS scores in WD patients. A substantial correlation was, however, identified between HAMD and PSQI (*r* = 0.338, *p* = 0.029), ESS (*r* = 0.320, *p* = 0.039), and PDSS (*r* = -0.471, *p* = 0.002) scores respectively.

**Table 2:** Correlations between sleep disturbance and psychopathological and Neurological variant and severity of the disease

		1	2	3	4	5	6	7
1. UWDRS	*r*	1.000						
	*P*	-						
2. NV	*r*	0.758	1.000					
	*P*	**< 0.001**	-					
3. HAMA	*r*	0.407	0.336	1.000				
	*P*	**0.008**	**0.030**	-				
4. HAMD	*r*	0.120	0.309	0.488	1.000			
	*P*	0.450	**0.046**	**0.001**	-			
5. PSQI	*r*	0.327	0.354	0.117	0.338	1.000		
	*P*	**0.035**	**0.021**	0.462	**0.029**	-		
6. ESS	*r*	-0.185	-0.037	0.000	0.320		1.000	
	*P*	0.241	0.815	0.998	**0.039**		-	
7. PDSS	*r*	-0.135	-0.236	-0.099	-0.360			1.000
	*P*	0.395	0.132	0.535	**0.019**			-

**Abbreviations:** Neurological variant (NV); Unified Wilson’s disease rating scale (UWDRS); Parkinson’s disease Sleep Scale (PDSS); Pittsburgh sleep quality index (PSQI); Epworth Sleepiness Scale (ESS); Hamilton Anxiety Scale (HAMA); Hamilton Depression Scale (HAMD)

Multiple linear regression analyses were carried out with PSQI, ESS, and PDSS scores as the dependent variable to further explore the significant factors influencing sleep quality. Three linear regression models revealed that HAMD was the significant determinant of PSQI (β = 0.686, *p* = 0.001), ESS (β = 0.410, *p* = 0.044), and PDSS (β = -0.624, *p* = 0.002) scores for the entire WD cohort (as displayed in Table 3).

**Table 3:** Linear regression models to predict sleep disturbance

	***F*** (***P***-value)	Adjusted ***R***^2^	Standard ***β***	***P***
**PSQI**	2.730 (0.044)	0.144		
UWDRS			0.026	0.889
NV			0.313	0.109
HAMA			-0.249	0.211
HAMD			0.410	**0.044**
**ESS**	4.123 (0.007)	0.234		
UWDRS			-0.162	0.361
NV			-0.032	0.862
HAMA			-0.356	0.062
HAMD			0.686	**0.001**
**PDSS**	4.283 (0.006)	0.243		
UWDRS			-0.040	0.412
NV			-0.149	0.820
HAMA			0.224	0.232
HAMD			-0.624	**0.002**

**Abbreviations:** Neurological variant (NV); Unified Wilson’s disease rating scale (UWDRS); Parkinson’s disease Sleep Scale (PDSS); Pittsburgh sleep quality index (PSQI); Epworth Sleepiness Scale (ESS); Hamilton Anxiety Scale (HAMA); Hamilton Depression Scale (HAMD)

## Discussion

As per our knowledge, we firstly comprehensively evaluated the clinical significance of depression, anxiety and sleep disturbance between Controls, NV, and NNV cohorts. The main findings were: (1) Depression, anxiety and sleep disturbance are frequently observed in severe forms in WD, particularly those with NV; (2) Statistical correlations were observed between depression and sleep disturbance in WD, where depression was the dominant determinant.

In our present work, WD subjects showed severe tremors on wakening and subtle extrapyramidal signs on neurological examination, supported by WD motor analysis [15]. The risk of tremors in WD patients may be linked to striatum damage. Furthermore, the severity and prevalence of sleep disturbance were elucidated by comparing validated scale scores with healthy controls [16, 17]. It was revealed that WD patients suffered from frequent sleep disturbance, mainly characterized by poor sleep quality, EDS, and difficulty staying asleep, consistent with the literature [17]. The WD patients present broad distribution of brain neuronal damage, including corpus striatum structural abnormalities and severe brain atrophy [18]. Neuroimaging studies have also found that copper deposits cause inhibition of dopamine transporter and D2 receptors in the striatum [19], indicating possible mechanisms associated with sleep disturbance. EDS may be caused by prolonged onset delay of sleep and rapid eye movements (REM) sleep, decreased sleep efficiency, and time [20], its pathogenic mechanisms may include an imbalance of neurotransmitters and damage to REM cells; however, this research lacked data for the objective auxiliary tests. In fact, only a few studies with video-polysomnography (vPSG) have objectively and in detail described sleep disturbance in WD, and Jernajczyk W professor concluded that vPSG should be recommended as an objective method to analyze sleep disturbance in WD [21]. They reported anxiety and depression more often than the healthy, facilitated by the patient’s response to the neurological deficits, physical incapacity, and chronic disease state. The literature suggests that WD individuals often exhibit mild cognitive disruption, which may contribute to and increase sleep disturbance in WD [22]. However, cognitive performance was not assessed in our cohort. The various psychiatric manifestations may be due to extensive and multiple systemic neuropathological alterations because of the disease.

It was revealed that the severity of anxiety, depression and sleep disturbance were markedly higher in the NV individuals than the NNV individuals. High T2 signals in the basal ganglia (BG), globus pallidus, striatum, and brain stem were usually observed in patients with neurological or psychiatric manifestations, as revealed by MRI analysis, explaining the present results [23]. ​In addition, brain MRI enables a semi-quantitative analysis of neuroradiological scale severity and sleep disturbance [24]. Specifically, the PDSS item scores, including difficulty staying asleep, were substantially elevated in the NV group than in the NNV subjects (*p* = 0.030), possibly because of metal accumulation and consequent brainstem neurodegeneration [25]. Difficulties in staying asleep were more severe in NV individuals, who were also more anxious and depressed than those among the NNV group, thus sleep disturbance may also be partially due to mood disorders.

The underlying mechanism and risk factors of sleep disturbance in WD remain to be fully determined. Many factors are involved in WD-associated insomnia: cirrhosis, lesions in the sleep-wake regulation system, nocturnal discomfort, psychosis, depression, anxiety, and medications. Here, UWDRS and NV were positively correlated with PSQI (*p* < 0.05), suggesting severe neurological symptoms in patients and worse sleep quality. Moreover, a statistical correlation was observed between HAMD and PSQI, ESS, and PDSS scores in WD subjects, respectively, confirming the severity and neurological variant of the disease, as well as the association of depression with sleep disturbance. To further analyze the determinants, multiple regression analysis was performed. Linear regression models indicated depression as the essential marker of sleep disturbance. Here, the hypothesis that depression could affect sleep disturbance in WD was assessed. As mentioned earlier, the metal deposition in the central nervous system’s BG might occur early; this could cause diverse psychiatric syndromes under diseased conditions [26]. By single-photon emission computed tomography, serotonergic deficits in WD were visualized. A decreased density of presynaptic serotonin transporters in the BG, thalamus, and hypothalamus was observed, indicating a potential mechanism [27].

The limitations of this investigation include the inevitable bias caused by the relatively small sample size; therefore, the interpretation of the results requires caution. As the gold objective independent validation of the instruments for WD, brain MRI and vPSG were not performed in all subjects and were not included in this research, and this aspect may represent a limitation of our study. It should be noted that the PDSS scale administered in this work was specifically developed to evaluate Parkinson’s disease patients, thereby limiting the generalizability of results.

## Conclusion

Depression, anxiety and sleep disturbance are frequently observed in severe forms in WD, particularly those with NV. Our investigation highlights that depression is an essential determinant of sleep disturbance and recommends depression screening and treatment for all WD patients. These symptoms of sleep disorder should be more systematically explored and studied.

## Data Availability

The datasets are available from the first author upon reasonable request.
